# Integrin and GPCR Crosstalk in the Regulation of ASM Contraction Signaling in Asthma

**DOI:** 10.1155/2012/341282

**Published:** 2012-09-29

**Authors:** Chun Ming Teoh, John Kit Chung Tam, Thai Tran

**Affiliations:** ^1^Department of Physiology, Yong Loo Lin School of Medicine, National University of Singapore, MD9, 2 Medical Drive, Singapore 117597; ^2^Department of Surgery, Yong Loo Lin School of Medicine, National University of Singapore, Singapore 119228

## Abstract

Airway hyperresponsiveness (AHR) is one of the cardinal features of asthma. Contraction of airway smooth muscle (ASM) cells that line the airway wall is thought to influence aspects of AHR, resulting in excessive narrowing or occlusion of the airway. ASM contraction is primarily controlled by agonists that bind G protein-coupled receptor (GPCR), which are expressed on ASM. Integrins also play a role in regulating ASM contraction signaling. As therapies for asthma are based on symptom relief, better understanding of the crosstalk between GPCRs and integrins holds good promise for the design of more effective therapies that target the underlying cellular and molecular mechanism that governs AHR. In this paper, we will review current knowledge about integrins and GPCRs in their regulation of ASM contraction signaling and discuss the emerging concept of crosstalk between the two and the implication of this crosstalk on the development of agents that target AHR.

## 1. Introduction

Airway hyperresponsiveness (AHR) is the exaggerated response to relatively low concentrations of constricting agents (such as methacholine or histamine) or indirectly acting stimuli (such as cold air, respiratory infections or allergens, exercise, or cigarette smoke) that is observed in asthmatic subjects [1]. Contraction of airway smooth muscle (ASM) cells that line the airway wall is thought to influence aspects of AHR, culminating in the generation of force and excessive narrowing or occlusion of the airway [2]. ASM contraction is primarily controlled by agonists that bind G protein-coupled receptors (GPCR), which are expressed on ASM. Studies have shown that the asthmatic airways can be completely occluded even with only 40% contraction of ASM cells following an asthma exacerbation to GPCR agonists, such as histamine, that induce muscle shortening [3, 4]. Therefore, ASM GPCRs are important targets for therapeutic agents in asthma treatment. However, there is increasing evidence to suggest that chronic use of *β*_2_-adrenergic receptor agonists, which act on GPCRs, is associated with worsening of bronchoconstrictor response to airway spasmogen [5], loss of asthma control, and exacerbation of asthma symptoms [6, 7], as well as an increased incidence of asthma-related morbidity and mortality [8]. Moreover, glucocorticoids, which are used as first line therapy for the treatment of inflammation associated with asthma, decrease AHR only if introduced early in disease diagnosis [9, 10]. Even then, there are side effects associated with the use of glucocorticoid when used at high dose and over long periods [10, 11]. Thus, the current treatment for asthma is based on symptom relief only and the ultimate goal of treating asthma is to target the underlying mechanisms, which include AHR.

We and others have shown that integrins may influence signaling events that contribute to AHR [12–14]. However, the mechanism behind this regulation remains to be fully elucidated. Moreover, there is increasing evidence to show that GPCRs interact with integrins to regulate ASM signaling pathways that are important in asthma. The cellular signaling processes include the regulation of cell adhesion, calcium signaling, injury and remodeling, mechanotransduction signaling and synaptic plasticity [15–18]. In this paper, we will review current knowledge about integrins and GPCRs in their regulation of ASM contraction signaling and discuss the emerging concept of crosstalk between the two and the implication of this crosstalk on the development of agents that target AHR.

## 2. Integrins and ASM Contraction Signaling

Integrins are heterodimeric transmembrane proteins comprising one *α* and *β* chain. The expression of different integrins in ASM, their potential ligands and change in expression in asthma are detailed in Table 1. Integrin activation via ECM protein binding leads to the formation of a complex called focal adhesion, which consists of many structural proteins such as vinculin, talin, *α*-actinin, and paxillin [19–21]. Integrins can signal through the cell membrane in both directions: inside-out signaling and outside-in signaling. The extracellular binding activity of integrins is regulated from the inside of the cell (inside-out signaling), while the binding of ECM proteins such as laminin elicit signals that are transmitted into the cell (outside-in signaling) [22]. It is through these signaling activation events that integrins regulate cell attachment, survival, proliferation, cell spreading, differentiation, cytoskeleton reorganization, cell shape, cell migration, gene expression, tumorigenicity, intracellular pH, and increase in concentration of cytosolic Ca^2+^ [23]. 

Activation of integrins by either contractile or mechanical stimuli can result in two signaling events to cause ASM cell contraction. Firstly, integrin activation causes the phosphorylation of focal adhesion kinase (FAK) and association with paxillin, leading to reorganization of the cytoskeleton [24–26]. Secondly, integrin activation will also increase intracellular Ca^2+^ concentration causing the phosphorylation of myosin light chain kinase (MLCK) and activation of myosin ATPase activity, and crossbridge cycling [24–26]. 

## 3. GPCR and ASM Contraction Signaling

GPCR spans the cell membrane seven times and transduces extracellular stimuli from the binding of cell surface ligands into intracellular second messengers. These second messengers are known as the heterotrimeric guanine nucleotide-binding protein (G proteins), which consists of G_*α*_, G_*β*_, and G_*γ*_ subunits [39]. G proteins bind to the intracellular domain of GPCR and transmit signals that are important in ASM cellular functions. These functions include regulation of ASM proliferation and secretion of cytokines, chemokines, eicosanoids, or growth factors that orchestrate airway inflammation and remodeling [40]. GPCRs are also implicated to play important role in ASM cell contraction. The regulation of ASM tone is mediated by a balance between G_q_- and G_s_-coupled signaling, with G_q_ being linked to ASM contraction signaling and G_s_ being linked to relaxation signaling [40–43]. Agonist binding causes the activation and association of GPCRs with G_q_, which promotes GTP binding and dissociation of G_*α*_ from G_*βγ*_ subunits. The dissociated G_q_ will then bind to effector phospholipase C, which then hydrolyses phosphoinositol 4,5-bisphosphate (PIP_2_) into 1,2-diacylglycerol (DAG) and inositol 1,4,5-triphosphate (IP_3_). The net effect of these events is to increase the levels of intracellular Ca^2+^ as well as to activate cell contractile machinery through Ca^2+^ and protein kinase C-(PKC-) dependent mechanisms [40]. Activated PKC is able to phosphorylate a number of substrates which include calponin [41]. Phosphorylated calponin loses its ability to inhibit actomyosin ATPase, which is required for ASM cell contraction [41]. 

## 4. Evidence for Integrin and GPCR Crosstalk

There is emerging interest in crosstalk between integrins and GPCRs (Table 2). For example, muscarinic agonists that bind G_12/13_ protein can induce FAK activation and autophosphorylation in Swiss 3T3 cells, a fibroblast cell line, which is associated with integrin engagement signaling [52]. Arg-Gly-Asp (RGD) is a consensus amino acid sequence found in ECM proteins that is recognized by integrins. It is found that muscarinic-induced FAK activation can be blocked by RGD peptide, suggesting crosstalk between GPCRs and integrins [52]. Similar observations have been observed for other GPCR agonists such as gastrin, endothelin, lysophosphatidic acid (LPA), angiotensin II, and bombesin [23, 53–56]. For example, stimulation of Swiss 3T3 cells with bombesin or endothelin results in FAK and paxillin phosphorylation and accompanied formation of focal adhesion plaques. This study suggests the formation of focal adhesion plaques as a common signal transduction pathway that mediates GPCR and integrin crosstalk. As for endothelial cells, angiotensin II is able to induce FAK and paxillin phosphorylation which results in augmented cell migration necessary for blood vessel repair and wound healing. This suggests a critical role for integrins in the angiogenic effect of angiotensin II via FAK activation. Taken together, the existence of distinct pathways leading to FAK activation suggests the possibility of synergistic interaction between GPCRs and integrin receptors. One of the key signaling events following integrin ligation is the activation of FAK. FAK activation recruits phosphatidylinositol-3-kinase (PI3K), leading to the activation of Akt that regulates cellular processes such as survival, proliferation, and contraction signaling [22]. Integrin-GPCR crosstalk is also linked with the activation of the mitogen activated protein kinase (MAPK) signaling pathway [55]. Lysophosphatidic acid and thrombin receptors alone can activate MAPK in PC12 cells and this was blocked by RGD peptide and cytochalasin D, which is an actin depolymerising agent involved in the remodeling of the cytoskeleton [55]. This data suggests important crosstalk between integrins and GPCRs in regulating MAPK signaling. Amin and coworkers show that *β*1 integrin plays a crucial role in negating the apoptotic effects of *β*-adrenergic receptor stimulation in cardiac myocytes via the involvement of FAK and PI3K/Akt pathways [57]. Furthermore, a nonreceptor tyrosine kinase, PYK2, is able to link GPCRs to focal adhesion-dependent ERK activation to provide a point of convergence between signaling pathways triggered by integrins and certain GPCR agonists (histamine) in HEK 293 (human embryonic kidney cell line) and HeLa Cells [58]. In another study, Short and coworkers show that the regulation of MAPK activity by integrins and P2Y class of G_q/11_-coupled receptors in human endothelial cells may involve activation of calcium and PKC [55]. Collectively, these studies support a role for integrin and GPCR crosstalk in physiological processes; however, integrin-GPCR interaction may be context-dependent given that different signaling mechanisms have been put forward.

The expression of ECM proteins can be regulated by GPCR ligands. For example, thrombin, sphingosine-1-phosphate, and LPA that signal through G_12/13_ and Rho A activation can increase the expression of the ECM protein Cyr61 (CCN1) in fibroblast, smooth muscle cells, and prostatic epithelial cells, respectively [44]. Cyr61 subsequently binds to integrin and activates downstream signaling pathways that regulate cell migration, survival, and proliferation. The engagement of integrin signaling pathway via GPCR ligands provide a means to amplify and sustain GPCR signaling in normal and pathophysiological cellular functions. In the asthma context, exaggerated GPCR signaling in AHR may contribute to increased expression of ECM proteins in the airway. The activation of integrins by these ECM proteins may thus amplify and sustain GPCR signaling to contribute to excessive bronchoconstriction that is observed in asthma exacerbations. 

Activated integrins organize supramolecular complexes consisting of cytoskeletal domains and associated receptors and signaling molecules that may contribute to the formation of specialized lipid microdomains, which are referred to as “lipid rafts” [59]. Until now, there is no evidence for integrin-mediated activation of heterotrimeric G protein signaling cascade outside lipid rafts. However, there is some evidence to show that ligation of integrins within supramolecular complexes can lead to activation of GPCRs. CD47, an integrin associated protein, forms complexes with *α*_V_ *β*_3_ integrin and activates G_i_ signaling [60]. Integrin association is also required for activation of G_o_ signaling by the P2Y_2_ receptor [45]. Recently, Berg and colleagues show that the relative amount of activated integrins at focal adhesion sites may govern signaling by *μ* opioid receptor, perhaps by altering interactions with G proteins [17]. Moreover, Lin and coworkers show that integrin ligation can trigger AMPA receptor-dependent Ca^2+^ influx and intracellular Ca^2+^ store release [61]. Taken together, crosstalk between integrins and GPCRs is relevant to ASM cells and possible in the asthma pathophysiological processes. 

**Figure 1:**
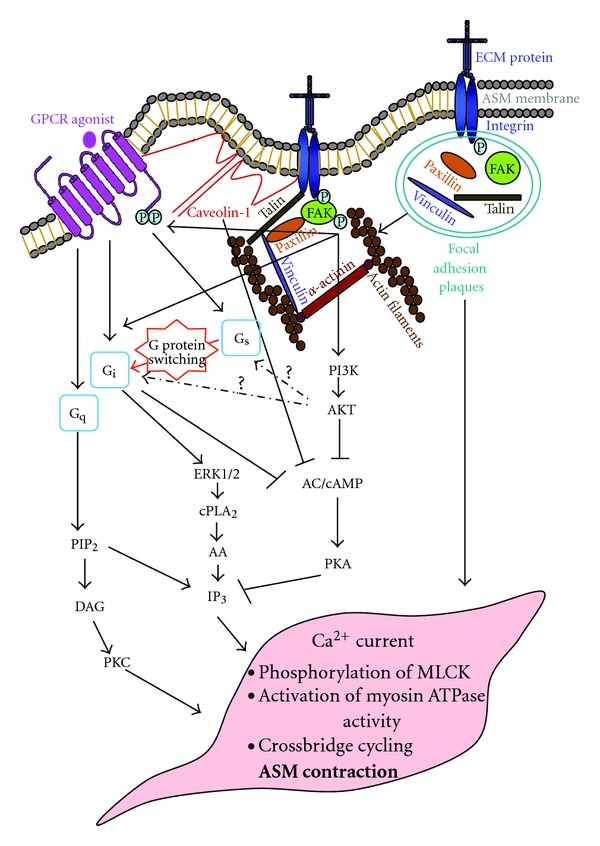
Schematic diagram showing the proposed crosstalk between integrins and GPCRs in ASM cell contraction signaling. Integrin activation is achieved via the formation of focal adhesion plaques leading to cytoskeleton reorganization, which is essential for actin polymerisation and recruitment of linker proteins for tension development. Integrin activation causes the phosphorylation of FAK and activation of downstream signaling events leading to ASM contraction. Integrin activation will also increase intracellular Ca^2+^ concentration to cause phosphorylation of MLCK and activation of myosin ATPase activity and crossbridge cycling. GPCR-induced ASM contraction signaling can be enhanced either by inhibition of cAMP/AC activity that regulates ASM relaxation signaling, or by activation of Ca^2+^ current that is necessary for ASM contraction signaling. Activation of integrins can attenuate GPCR-induced AC activity via the FAK/PI3K/Akt pathway. cAMP accumulation and AC activity can be decreased by integrin activation via G protein switching, in which G_i_ is activated instead of G_s_. Altered phosphorylation of GPCR by integrins is thought to underlie G protein switching in ASM cell. Caveolin-1 that binds integrin has been shown to regulate GPCR signaling. Caveolae which are rich in caveolin-1 function as negative regulators of cAMP accumulation in ASM cell. GPCR stimulation of Ca^2+^ can be enhanced by integrin via inhibition of cAMP/PKA and activation of the G_i_/ERK1/2/cPLA_2_/AA signaling. AA: arachidonic acid; AC: adenyl cyclase; AKT: protein kinase B; ASM: airway smooth muscle; cAMP: cyclic adenosine monophosphate; cPLA2: cytosolic phospholipase A2; DAG: diacylglycerol; ECM: extracellular matrix; ERK1/2: extracellular signal regulated kinase1/2; FAK: focal adhesion kinase; GPCR: G protein-coupled receptor; IP_3_: inositol 3,4,5-triphosphate; PIP_2_: phosphoinositol 4,5-bisphosphate; PI3K: phosphatidylinositol 3′-kinase; PKA: protein kinase A; PKC: protein kinas C.

There are currently limited studies regarding the involvement of both integrins and GPCRs in the regulation of ASM cell contraction in healthy and asthmatic condition (Figure 1). However, crosstalk between integrins and GPCRs in contraction signaling is evident in other cell types. In the context of cardiac muscle contraction signaling, Wang and colleagues show that laminin binding-*β*_1_ integrins in association with the actin cytoskeleton are able to attenuate adenylate cyclase (AC) activity. This in turn inhibits cholinergic regulation of L-type Ca^2+^ current in cardiac muscle contraction [62]. Subsequently, they also show that laminin binding-*β*1 integrins in conjunction with the actin cytoskeleton have the ability to reduce *β*1-adrenergic receptor-induced L-type Ca^2+^ and enhance *β*_2_-adrenergic receptor-induced L-type Ca^2+^ current in the same cell [63]. Recently, the same group shows that *β*_1_-integrin-induced activation of the FAK/PI3K/Akt pathway can inhibit *β*_1_-adrenergic receptor-mediated stimulation of L-type Ca^2+^ current in cardiac muscle contraction [64]. This study suggests that increased deposition of ECM proteins such as laminin in a failing heart may favor *β*_2_-adrenergic receptor signaling to *β*_1_-adrenergic receptor signaling, and this may be mediated in part via *β*1 integrin-induced FAK/PI3K/Akt pathway.

As for atrial myocytes, *β*_2_-adrenergic receptor stimulation of Ca^2+^ current is shown to be enhanced by *β*_1_ integrin via inhibition of cAMP/PKA and activation of G_i_/ERK/cPLA_2_/AA signaling [65]. This study suggests that increased ECM protein deposition in atrial diseases such as atrial fibrosis and/or hypertrophy may enhance *β*_2_-adrenergic signaling, which depends more on G_i_/ERK/cPLA_2_/AA signaling (contraction) instead of G_s_/AC/cAMP/PKA signaling (relaxation). Cheng and coworkers also elegantly show the relationship between *β*_1_ integrin and *β*-adrenergic receptor regulation of L-type Ca^2+^ current in neonatal rat ventricular myocytes [66]. Overexpression of *β*_1_ integrin impedes *β*-adrenergic receptor-induced Ca^2+^ current via inhibition of AC/cAMP activity [66]. Similar observation is also obtained in adult cat atrial myocytes [62]. These findings suggest an important role for integrin and *β*-adrenergic receptor crosstalk in a diseased heart in which it is associated with chronic overload of pressure, increased ECM proteins and integrin receptors. Remodeling of GPCR receptor functions in asthma may occur too as there is increased deposition of ECM proteins and altered expression of integrins in the asthmatic airways. Collectively, these studies suggest that integrin activation might play a role in GPCR-induced muscle contraction of the airways. 

In the context of ASM cell physiology there is only one study that links ECM proteins to GPCR-induced relaxation signaling [46]. Exposure of ASM cells to laminin decreases cAMP accumulation and AC activity [46]. The decrease in cAMP accumulation and AC activity could be due to a phenomenon known as “G protein switching” [46]. “G protein switching” occurs when agonists binding to the *β*_2_-adrenergic receptor leads to the activation of G_i_ rather than G_s_. The activation of G_i_ and decreased G_s_ signaling translate into low AC activity and thus decreased cAMP accumulation [46]. Altered phosphorylation states of the *β*_2_-adrenergic receptor may be the underlying cause of G protein switching [67]. Since integrins are able to phosphorylate cell surface receptors, it is thought to play a role in G protein switching [68]. Human ASM cells predominantly express AC isoforms V and VI. These isoforms can be inhibited by Ca^2+^ and G_i_ signaling but stimulated by PKC [69, 70]. As integrin activation leads to PKC activation and Ca^2+^ release and influx, it suggests that integrins may modulate AC activity. This would explain the decrease in AC activity of human ASM cells cultured on laminin [71, 72]. This finding is important given that cAMP and AC are regulators of ASM relaxation signaling and this is the first study to implicate that integrins may regulate ASM tone. However, the involvement of GPCR crosstalk with integrins in healthy and asthmatic ASM was not directly investigated in this study and future studies in this area are warranted.

GPCR signaling has been shown to be highly compartmentalized and disruption of this subcellular organization may affect GPCR function [73]. Integrin clustering is a crucial step towards the formation of focal adhesion. Focal adhesion is able to recruit various proteins that are involved in cell signaling cascades which include G proteins in GPCR signaling [74]. Contractile human ASM cells exhibit omega-shaped plasma invaginations known as caveolae (developed from lipid rafts that bind caveolin-1 protein) [75]. Caveolae associate preferentially with signaling proteins that have roles in controlling smooth muscle contraction signaling, for example, G_*α*_ protein, members of the Rho small GTPase family, and PKC [75]. Depending on the type of GPCR, upon ligand binding, receptors may remain, exit or translocate into caveolae [76–78]. Muscarinic M_3_ and histamine H_1_ receptors have been found within the caveolae enriched membrane fraction of human ASM [75]. Moreover, muscarinic M_3_ receptors and G_q_ protein cofractionate in caveolin-1 enriched ASM cell membranes [79]. Caveolin-1 is able to bind to integrin *α*-subunits and has been shown to regulate GPCR-mediated signaling [80, 81]. Caveolin-1 links integrin *α*-subunit to tyrosine kinase Fyn which then recruits Shc and Grb2. This sequence of events couples integrins to downstream signaling pathways such as Ras-ERK pathway. Caveolae function as negative regulators of cAMP accumulation. This suggests that integrin signaling regulated by caveolin-1 may serve as important modifier of GPCR signaling such as cAMP signaling in asthma. Caveolae are found in close proximity to peripheral sarcoplasmic reticulum and mitochondria, suggesting that caveolae may play a role in the spacial coordination of Ca^2+^-handling channels and organelles, which are implicated in ASM contraction signaling [82]. In addition, caveolae are anchored to the dystrophin glycoprotein complex (DGC). The DGC in turn links to ECM protein, laminin. This linkage is thought to help maintain membrane integrity [75, 83]. Collectively, these studies support the notion that caveolae may mediate ASM contractile response by aiding integrin and GPCR crosstalk signaling in asthma.

Integrins have also been implicated to regulate vascular smooth muscle cell contraction by mobilizing intracellular Ca^2+^. The addition of RGD peptide at millimolar range elicited increased levels of intracellular Ca^2+^ concentration [18]. This activation of ryanodine-sensitive Ca^2+^ store and lysosome-like organelles by RGD peptide [18, 84] suggests important role of integrin-dependent Ca^2+^ signaling in regulating smooth muscle contraction. In support, *α*_7_ *β*_1_ integrin has been implicated to regulate transient elevation of intracellular-free Ca^2+^ concentration from both IP_3_ evoked Ca^2+^ release from intracellular stores and extracellular Ca^2+^ influx through voltage-gated L-type Ca^2+^ channels in skeletal muscle cell [85]. 

Lastly, it is worth noting that GPCR agonists may promote ECM protein production, either directly, or indirectly by promoting autocrine TGF*β* release. TGF*β* is linked to thickening of ASM layer and deposition of collagen. Tatler and colleagues show that GPCR agonists, LPA and methacholine, induced TGF*β* activation via integrin *α*v*β*5 by ASM cells [38]. In support, Grainge and colleagues provide evidence that repeated bronchoconstriction with methacholine increases TGF*β* immunoreactivity within the airway epithelium and increases the thickness of the subepithelial collagen layer, which is indicative of an acute alteration in airway collagen dynamics [86]. These studies provide alternative means of crosstalk between GPCRs and integrins, and one that could amplify direct GPCR/integrin interactions.

## 5. Concluding Remarks

In summary, integrins may play a role in regulating GPCR-induced ASM cell contraction signaling in asthma. This finding may offer explanations for increased contractility of ASM cells in asthma in which ECM proteins and their binding receptor integrins are highly expressed. Thus, integrins may be an interesting therapeutic target to inhibit ASM contraction signaling in asthma. However, the development of integrin antagonists has proven to be challenging. The role of integrins in asthma is complex as multiple integrins may participate to exert asthma symptoms, making it difficult to specifically target integrins that are involved in ASM contraction signaling. Perhaps targeting “linker proteins” that link the crosstalk between integrins and GPCRs in ASM contraction signaling is a possible therapeutic strategy for the treatment of AHR in asthma. One such possible target is caveolin-1 that may regulate integrins and GPCRs crosstalk. Other possible targets may be those which participate in G protein switching that are induced by integrin activation. Nonetheless, further understanding of the mechanisms behind integrin and GPCR crosstalk in ASM cell contraction signaling will enhance the development of more tailored therapy in the future for asthma treatment where AHR is a feature.

## Figures and Tables

**Table 1:** Expression of different integrins in ASM, their potential ligands and change in expression in asthma.

Integrin	Expression in ASM	Potential ligands	Change in expression in asthma (human)	Reference
*α*1*β*1	Human, sheep, guinea pig	Collagen I, II, III, IV, laminin-111, fibronectin.	n.d.	[27–30]
*α*2*β*1	Human, guinea pig	Collagen I, IV, laminin-111, tenascin.	n.d.	[27–29, 31, 32]
*α*3*β*1	Human	Collagen I, fibronectin, laminin-211, laminin-221, laminin-322, laminin-511, laminin-521.	n.d.	[14, 27, 28]
*α*4*β*1	Human, sheep	Fibronectin, osteopontin, VCAM-1.	↑	[27, 28, 30, 33]
*α*5*β*1	Human, guinea pig	Fibronectin, osteopontin.	↑	[12, 27, 28, 32, 34, 35]
*α*6*β*1	Human	Laminin-111, laminin-411, laminin-511, laminin-521.	n.d.	[14, 28]
*α*6*β*4	Human	Laminin-322, laminin-511, laminin-521.	n.d.	[28]
*α*7*β*1	Human	Laminin-111, laminin-211, laminin-221.	n.d.	[14]
*α*8*β*1	Mouse	Fibronectin, tenascin, vitronectin	n.d.	[28]
*α*9*β*1	Human, guinea pig, mouse	ADAMs 1, 2, 3, 9, 15, factor XIII, L1-Cell adhesion molecule, osteopontin, tenascin, VCAM-1, von Willebrands factor.	↓	[28, 36, 37]
*α*v*β*1	Human	Fibronectin.	↑	[28, 32]
*α*v*β*3	Human	Fibrinogen, fibronectin, GSP, laminin, osteopontin, thrombospondin, vitronectin, von Willebrands factor.	n.d.	[27, 28, 32]
*α*v*β*5	Human, mouse	Osteopontin, vitronectin	↑	[38]

n.d.: not determined.

**Table 2:** Expression of ECM proteins/integrin ligands, their potential crosstalk with G proteins and change in expression in disease.

ECM/integrin ligands	Potential crosstalk with G proteins	Disease	Reference
Cyr61	G_12/13_	↑ in breast and endometrial cancers	[44]
RGD sequence in P2Y_2 _receptor	G_0_	n.d.	[45]
Laminin-111	G_i_, and G_s_	↑ in asthma	[46–48]
Fibronectin	G_q_ and G_12/13_	↑ in asthma	[47–50]
Collagen I	G_q_	↑ in asthma	[47, 48, 51]
Collagen V	G_i_ and G_s_	↑ in asthma	[46–48]

n.d.: not determined.
